# Should ethnicity be included in cardiovascular risk stratification?

**DOI:** 10.1007/s12471-014-0634-9

**Published:** 2014-11-19

**Authors:** B. J. Krenning, K. Van der Heiden

**Affiliations:** 1 Department of Cardiology, Thorax Centre Rotterdam, Erasmus Medical Center, PO Box 2040, 3000 CA Rotterdam, the Netherlands; 2 Havenziekenhuis, Rotterdam, the Netherlands; 3 Department of Biomedical Engineering, Erasmus Medical Center, PO Box 2040, 3000 CA Rotterdam, the Netherlands

Assessment of patients’ individual cardiovascular risk is common in routine clinical practice. However, the traditional risk factors to stratify risk are based on population estimates and may not directly apply to the individual patient. Patients with a high calculated cardiovascular risk may not suffer an event and vice versa. The Framingham score estimates the 10-year risk of a cardiovascular event based on several clinical and laboratory variables. Ethnicity is known to be related to risk of cardiovascular disease (CVD), but is not included in most risk scores. Also, differences in traditional risk factors do not fully explain the racial differences in CVD risk. To improve the ability to predict individual events, new biomarkers and imaging methods are currently being evaluated. Imaging for atherosclerosis using coronary artery calcium (CAC) and carotid intima media thickness (CIMT) may offer a valuable tool in this approach and is recommended (class IIa) in asymptomatic adults at moderate risk in the current ESC guidelines on cardiovascular disease prevention in clinical practice [1].

Multiple studies have evaluated the association of CAC scores with risk for adverse coronary events, but only a few studies have distinguished between subjects from different ethnic groups. In this issue of the Netherlands Heart Journal, Erqou et al. [2] assessed the racial differences in the burden of CAC and CIMT between Blacks and Whites using data from an on-going community-based prospective cohort study (heart SCORE). The authors conclude that Black race is independently associated with greater CIMT but less CAC than White race, also after adjustment for traditional risk factors.

A correlation between ethnicity, CAC, and events was previously reported in the Multi-Ethnic Study of Atherosclerosis (MESA), a large prospective, population-based study of samples from six urban communities [3, 4]. It was reported that the prevalence and extent of coronary calcification differ substantially among ethnic groups [3]. In the MESA study, CAC was measured to predict coronary events in four major ethnic groups [4]. After a median follow-up of nearly 4 years, a strong correlation was found between calcium scores and coronary risk. Adjusting for standard risk factors, a doubling of the CAC score was associated with a 25 % increase in the risk for a major event. However, the association was independent of ethnicity. Unfortunately, Erqou et al. [2] do not present data on cardiovascular events to relate their results to future cardiovascular events.

The novelty of the work by Erqou et al. lies in the additional biochemical marker analysis assessment. Six markers were analysed, i.e. small-dense low-density lipoprotein (sdLDL), high-sensitivity C-reactive protein (hsCRP), interleukin-6 (IL-6), soluble intercellular adhesion molecule 1 (sICAM-1), CD40 ligand (CD40L), and endostatin. Small-dense LDL was positively associated with increased CIMT (similar association among Blacks and Whites) and IL-6 concentration was positively associated with higher CAC among Blacks (trend in Whites). IL-6 is a promising biomarker for linking inflammation to future vascular events [5]. As the authors suggest, the low CAC and high IL-6 in the Black population might reflect less calcified coronary lesions and a higher inflammatory state and therefore a patient at risk for future cardiac events. This could, hypothetically, be associated with the higher risk of CVD in Blacks. Further studies are needed to confirm such a difference in atherosclerotic pathophysiology between ethnic groups.

In conclusion, this study confirms the earlier findings that CAC and CIMT values differ among ethnic groups and points to specific biomarkers that might be used to create race-specific algorithms for risk stratification. Hopefully, in the near future, we may further determine how these tests can be used to achieve better outcomes for individual patients.
